# A study on the recognition of monkeypox infection based on deep convolutional neural networks

**DOI:** 10.3389/fimmu.2023.1225557

**Published:** 2023-12-07

**Authors:** Junkang Chen, Junying Han

**Affiliations:** College of Information Science and Technology, Gansu Agricultural University, Lanzhou, China

**Keywords:** monkeypox images, residual convolutional networks, deep learning, aided diagnosis, contextual transformer

## Abstract

**Introduction:**

The World Health Organization (WHO) has assessed the global public risk of monkeypox as moderate, and 71 WHO member countries have reported more than 14,000 cases of monkeypox infection. At present, the identification of clinical symptoms of monkeypox mainly depends on traditional medical means, which has the problems of low detection efficiency and high detection cost. The deep learning algorithm is excellent in image recognition and can extract and recognize image features quickly and reliably.

**Methods:**

Therefore, this paper proposes a residual convolutional neural network based on the λ function and contextual transformer (LaCTResNet) for the image recognition of monkeypox cases.

**Results:**

The average recognition accuracy of the neural network model is 91.85%, which is 15.82% higher than that of the baseline model ResNet50 and better than the classical convolutional neural networks models such as AlexNet, VGG16, Inception-V3, and EfficientNet-B5.

**Discussion:**

This method realizes high-precision identification of skin symptoms of the monkeypox virus to provide a fast and reliable auxiliary diagnosis method for monkeypox cases for front-line medical staff.

## 1 Introduction

According to the World Health Organization (WHO) epidemic records, in 1958, the pathogen “monkeypox virus” of Macaca cynomolgus monkeys was first identified in the laboratory of Copenhagen, Denmark (1). In 1970, the first human infection with the monkeypox virus was found in the Democratic Republic of Congo. Since then, monkeypox has been prevalent in Central and West African countries. In 2003, the United States reported the first outbreak of monkeypox outside Africa. At the beginning of May 2022, the spread of the monkeypox virus was first discovered in Britain, and then there were more than 100 cases of monkeypox, and there was a phenomenon of community transmission. Then, monkeypox cases were reported in the United States, Portugal, Spain, Canada, Belgium, Sweden, Italy, and other countries (2). From January 2022 to January 2023, WHO reported 84,733 laboratory-confirmed cases in 110 countries and regions, including 80 deaths.

Monkeypox virus (MPXV) is an enveloped double-stranded DNA virus that belongs to the genus Orthopoxvirus of Poxviridae, together with Variola virus (VARA) and Cowpox virus (CPXV) (3). Monkeypox is a zoonotic disease, and African rodents are the primary hosts of the monkeypox virus. Its infection route is similar to smallpox, and it can be spread through respiratory droplets, body fluids, infected animals, or articles contaminated by infected people (4, 5). After humans are infected with the monkeypox virus, the incubation period is usually 7~14 days, and the longest is 21 days. Then entering the prodromal stage, there will be prodromal symptoms such as lymph node enlargement, fever, headache, and muscle pain, which generally last for 1~2 days (6); Finally, it enters the eruption period, when the patient is highly contagious, that is, the period of high probability of infection, during which it is most dangerous for uninfected people to contact the patient. History has proved that unidentified or misdiagnosed infectious diseases are the decisive factors for super-transmission events (7). Therefore, it is urgent to strengthen the global emergency response-ability to public health events and efficiently provide reliable diagnostic information for patients suspected of monkeypox infection.

Currently, the diagnosis of monkeypox cases mainly depends on traditional medical equipment and the artificial experience judgment of doctors. For example, medical and health institutions detect the sequence-specific DNA sequence of the monkeypox virus by Polymerase Chain Reaction (PCR) and analyze its structure and function or isolate the monkeypox virus from clinical and animal specimens by cell culture (8). With the unremitting efforts of researchers, laboratory detection technology has made a breakthrough. Lv et al. (9) proposed a loop-mediated isothermal amplification (LAMP) for detecting the monkeypox virus, and its sensitivity was about ten times higher than that of standard PCR. The above method is a direct biochemical analysis of the monkeypox virus. Although the experimental results are highly reliable, the whole operation process has strict requirements on laboratory grade, high-risk factors, and long detection time, which is not conducive to investigating suspected monkeypox virus infection on a large scale. Moreover, most patients are in the eruption stage at the time of initial diagnosis, and their transmission risk is the highest, which will seriously affect the personal safety of doctors.

In recent years, with the updated iteration of computer vision technology and hardware equipment, image recognition using deep learning algorithms has become the mainstream method, and it has also achieved good results in disease investigation (10–13). The experimental sample is usually a two-dimensional picture of the diseased part taken by medical equipment, which avoids the close contact between medical staff and patients as much as possible and dramatically guarantees the safety of front-line workers. The related research of deep learning technology in auxiliary medical diagnosis includes El Asnaoui et al. (14) using various CNN models to rapidly diagnose novel coronavirus, among which the recognition accuracy of the models exceeds 96%. Ardakani et al. (15) evaluated ten deep-learning models using a small data set, including 108 COVID-19 patients and 86 non-COVID-19 patients, and achieved 99% accuracy. Prellberg et al. (16) used the ResNeXt network to efficiently classify the microscopic images of white blood cells, and the F1 score reached 88.89%. Feng Wang et al. (17) designed a method based on CNN to differentiate and diagnose benign and malignant nodules in lung CT images and predict the degree of malignancy, and all indicators showed promising results. Roy et al. (18) used different segmentation techniques to detect skin diseases like acne, candidiasis, cellulitis, chickenpox, etc. Ahsan et al. (19) used the improved VGG16 model to detect monkeypox. Ali et al. (20) used VGG16, ResNet50, and Inception-V3 to classify monkeypox and other diseases, among which ResNet50 achieved the best overall accuracy of 82.96%. Mohbey et al. (21) presenting a hybrid technique based on Convolutional Neural Networks (CNN) and Long Short-Term Memory Networks (LSTM). In this study a knowledge graph of related events based on Twitter data, which provides a real-time and eventful source of new information. The recommended model’s accuracy was 94% on the monkeypox tweet dataset. The findings of this research contribute to an increased awareness of monkeypox infection in the general population. Diponkor et al. (22) proposed an improved model MonkeyNet based on DenseNet-201, which classifies monkeypox from various skin images, and implements the model in a reliable mobile application, and really supports the diagnosis of medical staff. The above research aims to show that deep learning is effective in the medical field and the auxiliary diagnosis of skin infection symptoms of viruses, which can improve disease diagnosis efficiency.

The remarkable achievements of the above-mentioned deep learning technology in disease detection provide a solid basis for using deep learning technology to recognize the image of monkeypox cases. Therefore, this paper proposes a residual convolutional neural network based on the λ function and contextual transformer (LaCTResNet) for the image recognition of monkeypox cases. The series of network models are tested on an independent test set of monkeypox images, and the recognition accuracy of the optimal model reaches 91.85%, which is 15.82%, 7.79%, and 29.89% higher than that of the benchmark models ResNet50, CoTResNet50, and LambdaResNet50, respectively. Compared with the similar models AlexNet, VGG16, Inception-V3, and EfficientNet-B5, the recognition accuracy is improved by 25.03%, 20.66%, 34.00%, and 16.05%, respectively. The experimental results fully prove the feasibility and effectiveness of this method in clinical image recognition of monkeypox skin infection to provide a low-cost, high-efficiency, safe, and reliable auxiliary diagnosis method for medical personnel.

The following is the main work of this study:

- First, to address the problems of the latest monkeypox public dataset (which consists of images from six different categories, including Monkeypox, Varicella, Cowpox, Measles, Variola, and Health images.) on the Kaggle platform, which suffers from the blurring of some of the images as well as the reoccurrence of the images with high similarity, we manually filtered the dataset and utilized the data enhancement strategy to construct a reliable monkeypox dataset.
- Secondly, for the weak ability of traditional convolutional layers for sequence modeling and the lack of ability to deal with long-distance dependencies, we introduce the λ function layer and CoT function layer and define the residual convolution module based on λ function as well as the residual convolution module based on contextual transformer. The optimal collocation form of the two new modules is derived after many experiments, and the optimal collocation ratio of the backbone modules is derived on this basis. Our proposed model has higher recognition accuracy and can assist medical workers in diagnosing and treating more safely and efficiently.
- Finally, the optimal model proposed in this study is compared with the benchmark model and the classical model based on the same conditions. The model’s performance is evaluated from all aspects and multiple perspectives through metrics such as precision, recall, F1 score, and AUC score, and finally, it is concluded that the model proposed in this study has a more excellent recognition performance.

The following section is organized: details of the experimental dataset are given in Section 2. After that, Section 3 presents the design ideas of the proposed model, the overall architecture, the algorithmic ideas of the residual convolution module based on the λ function, and the residual convolution module based on the contextual transformer. Section 4 describes the experimental equipment setup hyperparameter settings and presents the data from the ablation and comparison experiments. Section 5 presents the evaluation metrics of the model and provides a comprehensive and objective discussion and analysis of the experimental results of the model on the test set. Section 6 briefly summarizes the work process and results of this study.

## 2 Dataset

**Figure 1:**
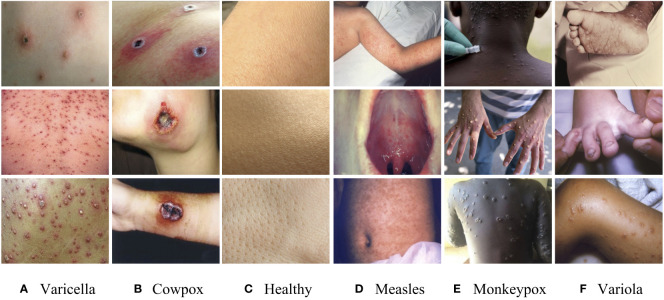
Sample images of the dataset. **(A)** Varicella, **(B)** Cowpox, **(C)** Healthy, **(D)** Measles, **(E)** Monkeypox, **(F)** Variola.

The dataset of the monkeypox in this study mainly comes from the Kaggle platform, which has attracted the attention of 800,000 data scientists. In order to ensure the authenticity and validity of the data, we invited immunologists to identify the image data set one by one, and it further confirmed the authenticity of the image data and the accuracy of the disease categories. The symptoms of various viruses in the monkeypox data set are shown in **Figure 1**.

The above figure shows that these viruses have seriously eroded the skin, and the differences in symptoms are not noticeable. It is easy for doctors to make misdiagnoses only by naked eye observation in the initial diagnosis. Similarly, in the training of the model, it will also lead to misjudgment due to the high similarity of image features. Therefore, we enhanced the original data set and adjusted the image’s brightness, contrast, and position information. The final image data set includes 4,235 clinical images of five kinds of virus infections (Varicella, Cowpox, Measles, Monkeypox, and Variola) and healthy skin images. In order to avoid the uneven distribution of image data caused by subjective interference when manually dividing the data set, we use a random screening algorithm to separate the images in the data set. Each case category is divided into training sets, verification sets, and test sets according to the ratio of 6:2:2 to ensure mutual independence between images. The case categories and the number of images included in the data set are shown in **Table 1**. This study focuses on distinguishing monkeypox from other types of cases. However, the five different types of cases are not classified into one category to cope with the situation that the monkeypox virus may mutate from two types to multiple types in the future (refer to the multiple mutations in Covid-19, which will lead to more violent global epidemic transmission events). Therefore, we individually divide the remaining five categories to train a model with solid robustness and generalization.

**Table 1:** The details of the dataset.

Classification	Before	After
Varicella	178	890
Cowpox	54	270
Healthy	50	250
Measles	47	235
Monkeypox	160	800
Variola	358	1790
**Total**	847	4235

## 3 The proposed mode

### 3.1 The design idea of the backbone

**Figure 2:**
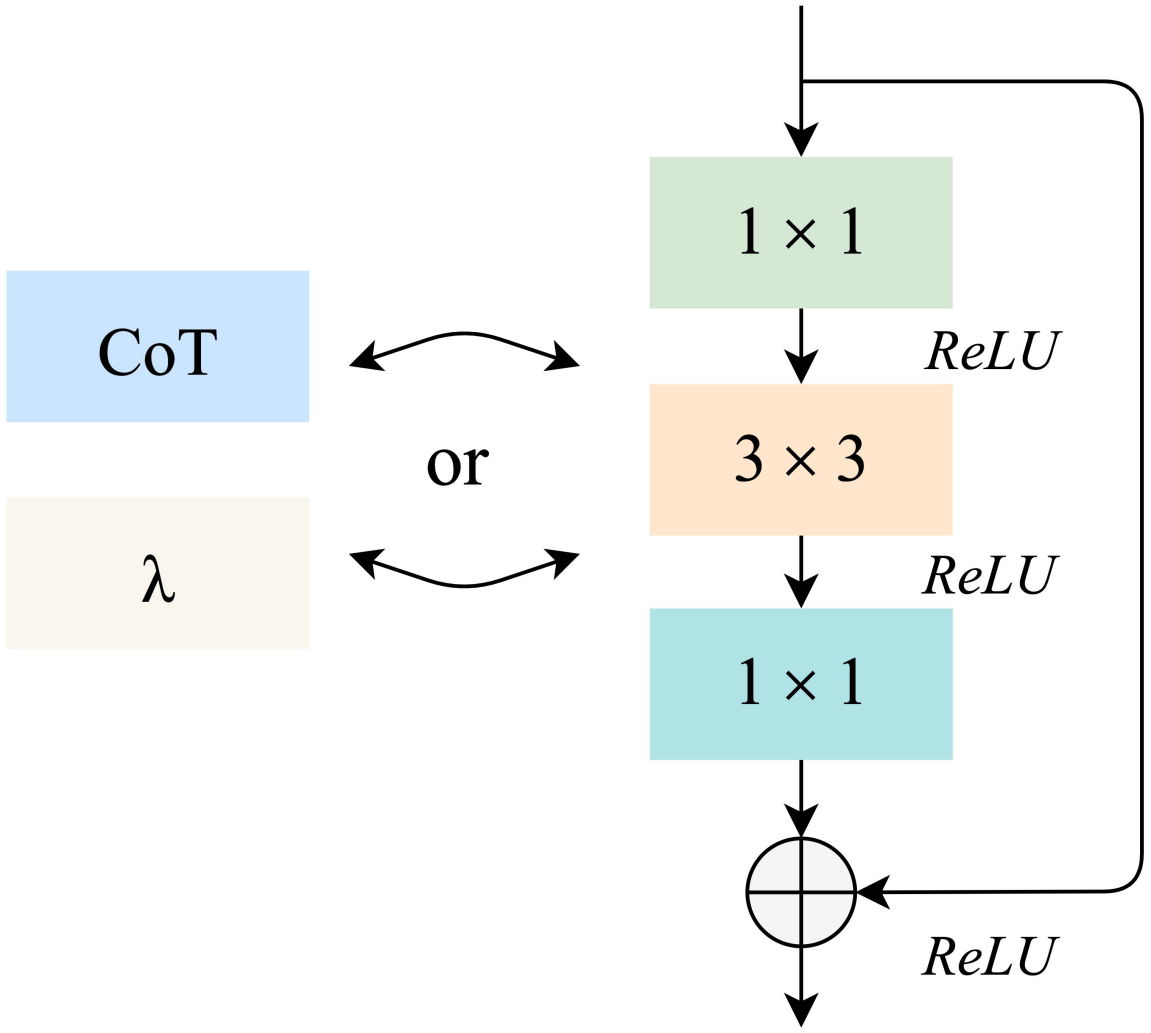
The design idea of the backbone.

To meet the requirements of high efficiency and high precision in monkeypox case identification, we define a residual convolution module based on the λ function and a residual convolution module based on the contextual transformer, as shown in **Figure 2**. The design idea is to replace the 3×3 convolution layer in the traditional residual module with λ calculation layer and CoT calculation layer. These two computing layers will be detailed in sections 3.3 and 3.4.

**Figure 3:**
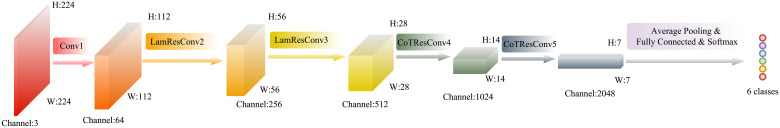
The overall architecture of the LaCTResNet model. The cube represents the output tensor; H, W, and Channel represent the height, width, and number of channels of the tensor, respectively. The circle represents the output category.

In this paper, LaCTResNet neural network model is defined by improving the feature extraction module of the ResNet backbone network, and the overall framework of the network model is shown in **Figure 3**. For the infected image of the monkeypox virus to be identified, this network finally outputs the category of infected virus predicted by the network model after passing through a convolution layer, two residual convolution modules based on the λ function, two residual convolution modules of the contextual transformer, an average pooling layer, a fully connected layer, and a Softmax layer. In the figure, Conv1 represents the ordinary convolution layer, LamResConv2 and LamResConv3 represent the λ function residual convolution module, and CoTResConv4 and CoTResConv5 represent the contextual transformer residual convolution module.

### 3.2 The principle of residual learning

**Figure 4:**
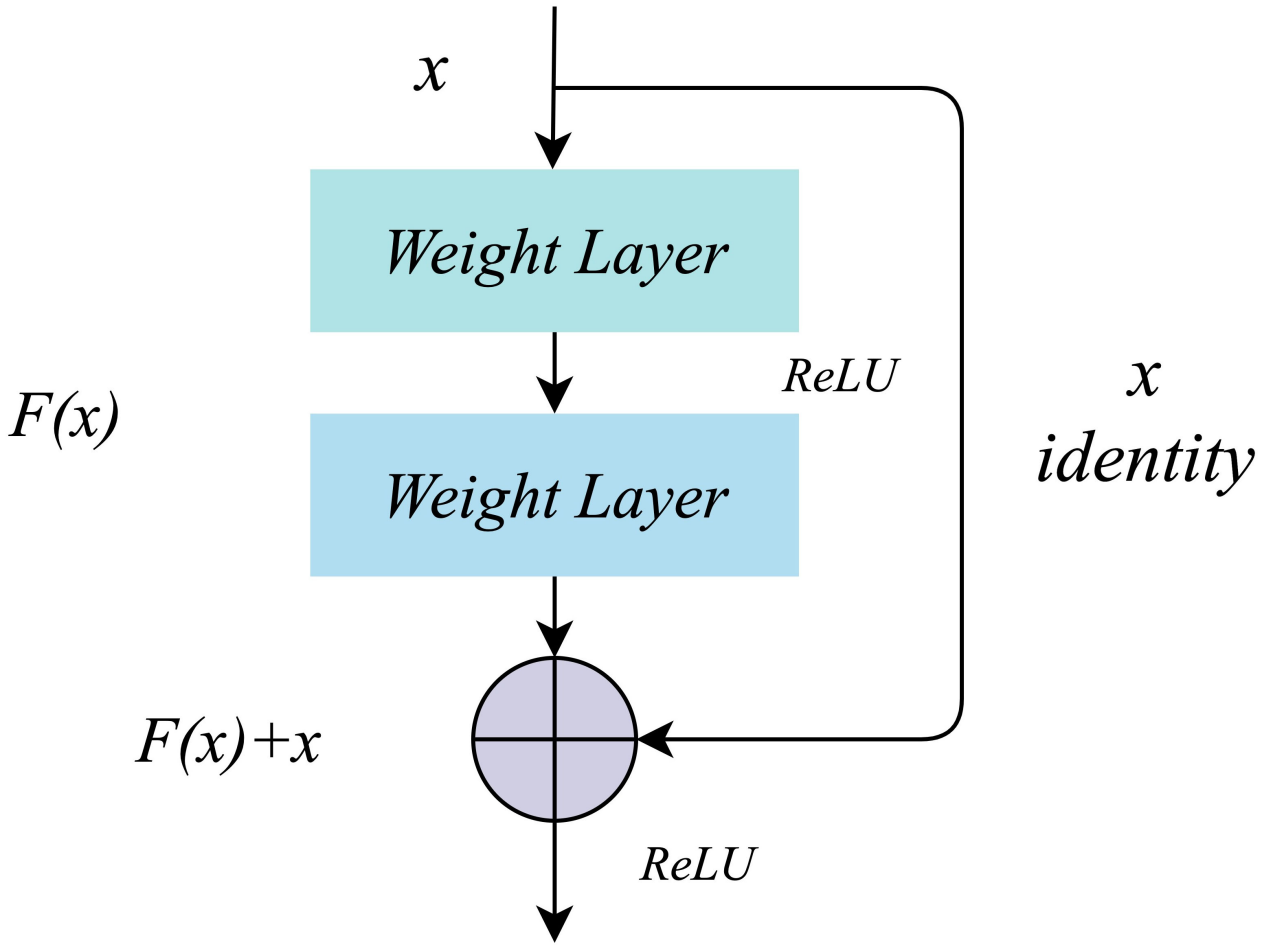
Residual structure.

The working mechanism of residual learning (23) is realized by residual connection. There are no redundant branches in the traditional convolutional neural network. From top to bottom, the input signal will be transformed nonlinearly at each layer and directly transmitted to the next layer. The structure of residual learning includes the main branch and the residual difference branch, as shown in **Figure 4**. The input signal *x* enters the main branch, passes through the *Weight Layer* and the activation function *ReLU*, and outputs the signal *F(x)*; The residual differential branch directly copies the input signal *x* and transmits it to the output end of the main branch through the cross-layer residual connection, and adds it with the output signal *F(x)* of the main branch to get *F(x)+x*, which is transmitted to the next layer after the activation function *ReLU*. Through residual connection, the main branch’s output signal contains the characteristics of traditional nonlinear transformation and residual characteristics. The residual feature represents the difference between the input signal and the expected output. By adding it to the output signal of the main branch, the network can learn these differences more efficiently, improve the accuracy of network feature extraction and recognition, and effectively avoid network degradation.

### 3.3 Residual convolution module based on λ function

**Figure 5:**
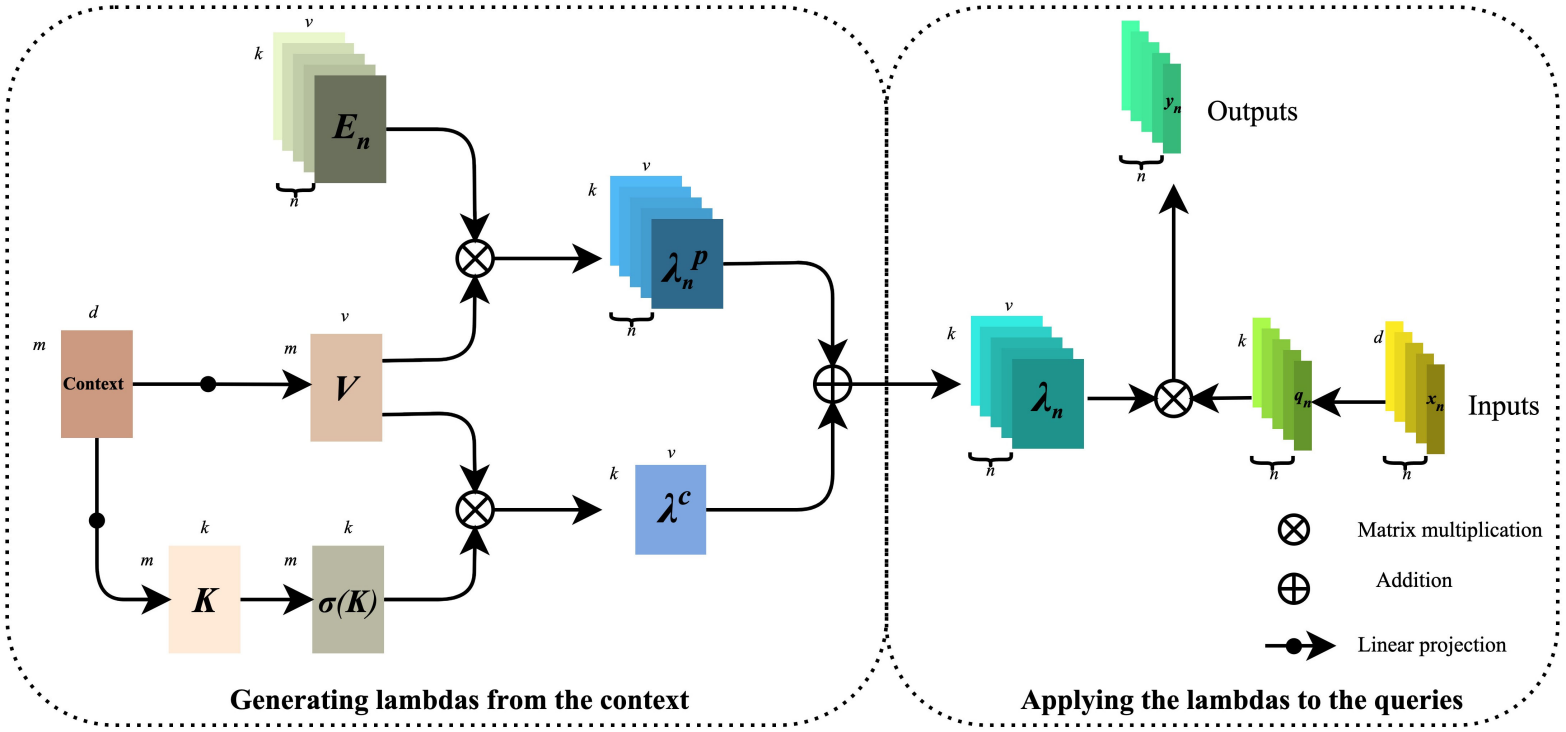
Calculation diagram of the λ function layer.

Modeling long-distance information interaction is an essential topic in deep learning. At present, the mainstream paradigm is the attention mechanism. It is easy to find that the high occupation of secondary memory is not conducive to dealing with long sequences or multi-dimensional input by analyzing the calculation diagram of the attention mechanism. Considering the limitation of self-attention, we introduce the λ function layer (24) into the model, which provides a novel general framework for capturing long-range information interaction between input signals and structured contexts. The λ function layer captures the information interaction by transforming the available context into a linear function λ and applying these linear functions to each input value respectively. The attention mechanism defines a convolution kernel of similarity between input and context. At the same time, the λ function layer aggregates the context information into a linear function with a fixed size, thus skillfully solving the situation that attention tries to occupy much memory. The detailed calculation diagram of the λ function layer is shown in **Figure 5**. The left part is λ function generated by context, and the right amount is λ function applied to the query.

From the above information, we can draw that, firstly, the Context gets *V*(Value) and *K*(Key) through linear projection, and the mathematical expressions are shown in formulas (1) and (2):


(1) $$K=CW_{K}\in {\mathbb{R}}^{\left|m\right|\times \left|k\right|},$$

(2) $$V=CW_{V}\in {\mathbb{R}}^{\left|m\right|\times \left|v\right|}$$

Next, *V* is multiplied with the position code *E_n_* to obtain the position-based $\lambda_{n}^{p}$ , and the mathematical expression is shown in equation (3):


(3) $$\lambda_{n}^{p}=E_{n}^{T}V\in {\mathbb{R}}^{\left|k\right|\times \left|v\right|},E_{n}\in {\mathbb{R}}^{\left|m\right|\times \left|k\right|},$$

*K* is then obtained by the normalization operation $\bar{K}$ with the mathematical expression shown in equation (4):


(4) $$\bar{K}=\sigma \left(K\right)=softmax\left(K,axis=m\right)$$

After that, $\bar{K}$ is multiplied with *V* to obtain the content-based $\lambda_{c}$ with the mathematical expression shown in equation (5):


(5) $$\lambda_{c}={\bar{K}}^{T}V\in {\mathbb{R}}^{\left|k\right|\times \left|v\right|}$$

Finally, $\lambda_{c}$ and $\lambda_{n}^{p}$ are multiplied to obtain $\lambda_{n}$ containing location and context information, and the mathematical expression is shown in equation (6):


(6) $$\lambda_{n}={\bar{K}}^{T}V+E_{n}^{T}V\in R^{\left|k\right|\times \left|v\right|}$$

The right part is to map the input value *X* (*X* $\in \left\{x_{1},x_{2},x_{3},\cdots ,x_{n}\right\}$ ), by features to *Q*(Query), *Q* $\in \left\{q_{1},q_{2},q_{3},\cdots ,q_{n}\right\}$ . Then, $\lambda_{n}$ ( $\lambda_{n}\in \left\{\lambda_{1},\lambda_{2},\lambda_{3},\cdots ,\lambda_{n}\right\}$ ) on the left side is applied to *Q* one by one. The final output *Y* (*Y* $\in \left\{y_{1},y_{2},y_{3},\cdots ,y_{n}\right\}$ ) is mathematically expressed as shown in equations (7) and (8):


(7) $$Q=XW_{Q}\in {\mathbb{R}}^{\left|n\right|\times \left|k\right|},$$

(8) $$y_{n}=\lambda_{n}^{T}q_{n}={\left(\lambda_{c}+\lambda_{n}^{p}\right)}^{T}q_{n}\in {\mathbb{R}}^{\left|v\right|},$$

### 3.4 Residual convolution module based on contextual transformer

**Figure 6:**
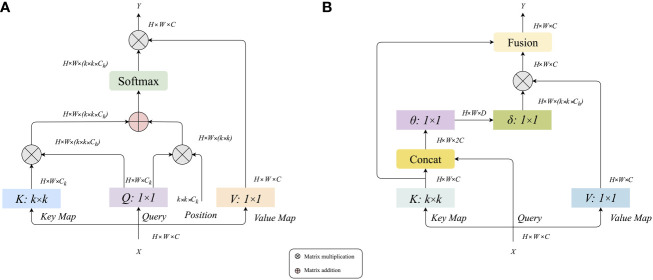
**(A)** Calculation diagram of traditional attention mechanism, **(B)** Calculation diagram of contextual transformer layer.

**Figure 6A** shows that the traditional self-attention can trigger feature interaction in different spatial positions. However, all the attention matrices of *Query* and *Key* are realized by calculating independent query key pairs, ignoring the rich context information between keys, thus limiting the visual learning ability of the self-attention mechanism in two-dimensional images. Therefore, we introduce the Contextual Transformer Layer (CoT) (25) into the traditional residual module and construct a residual convolution module based on contextual transformation, the structure of which is shown in **Figure 6B**. Through the fusion modeling of local static context and dynamic global context, the CoT computing layer can enlarge the feature distance between samples, improve the problems of significant parameters and weak feature extraction ability in the original ResNet network, and realize the effective extraction of feature information.

From the information in Figure (b), it can be seen that the input feature X of size *H × W × C* is given, and three variables *Q = X*, *K = X*, and *V = XW_V_* are defined (here only *V* is mapped to the feature, and the original *X* values are still used for *Q* and *K*). A grouped convolution of *k × k* (the value of *k* is taken as 3 in this experiment) is performed on *K* to obtain *K* with local context information representation (denoted as *K^1^*, *K^1^* ∈*R^H×W×C^*), and this *K^1^* can be seen as static modeling on the local information. Then *K^1^* and *Q* were Concat, and the result of Concat was subjected to two successive *1×1* convolution operations to obtain the attention matrix *A*. The mathematical expression is shown in equation (9):


(9) $$A=\left[K^{1},Q\right]W_{\theta }W_{\delta }$$

Unlike the traditional self-attentive mechanism, the A-matrix here is obtained from the interaction of Query information and local context information *K^1^*, rather than just a simple correspondence between Query and Key. That is, the guidance of regional context modeling enhances the self-attention mechanism. This attentional computational graph *A* and *V* are then subjected to a matrix product operation, which yields *K^2^* for dynamic context modeling, with the mathematical expression shown in equation (10):


(10) $$K^{2}=V\times A,$$

Then the final feature result *Y* is obtained by fusing *K^1^* from local static context modeling and *K^2^* from global dynamic context modeling. The design of the CoT layer unifies the context mining between adjacent keys and the self-attentive learning of 2D feature maps, using the context information between input keys to guide the self-attentive learning, thus avoiding the introduction of extra branches for context mining and improving the representational power of the network.

## 4 Experiment

### 4.1 Configuration of experimental environment

We conducted experiments on a computer with a GPU model NVIDIA GeForce GTX 3090, and the details are shown in **Table 2**.

**Table 2:** Experimental parameters and configuration.

Name	Configuration Information
Operating System	Ubuntu 18.04
CPU	Intel(R) Xeon(R) CPU E5-2680 v4 @2.40GHz (7 Cores)
RAM	30 GB
GPU	NVIDIA GeForce GTX 3090
Video Memory	24 GB
Code management software	PyCharm Community 2021.1.1
Computer Language	Python 3.8
Deep learning framework	PyTorch 1.9.0
Learning Rate	0.001
Batch Size	16
Epoch	100
Loss Function	Cross Entropy Loss
Optimization Algorithm	SGD

### 4.2 Ablation experiments

Firstly, the ratio of four groups of residual convolution modules is fixed at 3:4:6:3. That is, the module configuration ratio of ResNet50 is followed. Then, the 3×3 convolution in four groups of traditional residual convolution modules is replaced by the λ function layer or CoT layer for discussion. A total of five groups of experiments were carried out, and there were sixteen situations: in the first group of experiments, all four groups were replaced by the CoT layer; in The second group of experiments, replacing one group with λ function layer and the other three groups with CoT layer, has four situations; The third group of experiments, replacing two of them with λ function layer and the other two with CoT layer, has six situations; The fourth group of experiments, replacing three of them with λ function layer and the other with CoT layer, has four situations; In the fifth group of experiments, all four groups were replaced by λ function layer. The experimental results are shown in **Table 3**.

**Table 3:** The recognition accuracy and parameters of models with different types of residual modules on monkeypox independent test set.

Group	Model Name	λ Function Layer	CoT Layer	Accuracy	Params (M)
1	CoTResNet50	[0, 0, 0, 0]	[1, 1, 1, 1]	84.06%	33.79
2	LaCTResNet5001	[1, 0, 0, 0]	[0, 1, 1, 1]	87.37%	33.61
	LaCTResNet5002	[0, 1, 0, 0]	[1, 0, 1, 1]	87.25%	32.82
	LaCTResNet5003	[0, 0, 1, 0]	[1, 1, 0, 1]	83.94%	27.89
	LaCTResNet5004	[0, 0, 0, 1]	[1, 1, 1, 0]	82.29%	21.90
3	LaCTResNet5012	[1, 1, 0, 0]	[0, 0, 1, 1]	89.02%	32.65
	LaCTResNet5013	[1, 0, 1, 0]	[1, 0, 0, 1]	84.89%	27.72
	LaCTResNet5014	[1, 0, 0, 1]	[0, 1, 1, 0]	86.19%	21.72
	LaCTResNet5023	[0, 1, 1, 0]	[1, 0, 0, 1]	79.69%	26.93
	LaCTResNet5024	[0, 1, 0, 1]	[1, 0, 1, 0]	84.53%	20.93
	LaCTResNet5034	[0, 0, 1, 1]	[1, 1, 0, 0]	68.71%	16.00
4	LaCTResNet50123	[1, 1, 1, 0]	[0, 0, 0, 1]	83.00%	26.75
	LaCTResNet50124	[1, 1, 0, 1]	[0, 0, 1, 0]	88.43%	20.76
	LaCTResNet50134	[1, 0, 1, 1]	[0, 1, 0, 0]	76.86%	15.83
	LaCTResNet50234	[0, 1, 1, 1]	[1, 0, 0, 0]	62.34%	15.04
5	LambdaResNet50	[1, 1, 1, 1]	[0, 0, 0, 0]	61.98%	14.86

Looking at the above table, we can see that the effect of replacing all four groups of modules with the λ function layer is not ideal, and the recognition accuracy is only 61.98%; The CoT layer replaces all of them, and the recognition accuracy is 84.06%. In the second group of experiments, the best effect is that the λ function layer replaces the first group, and the other three groups are the CoT layer, and the recognition accuracy reaches 87.37%. In the third group of experiments, the best effect is that the λ function layer replaces the first and second groups, and the CoT layer replaces the other two groups, and the recognition accuracy reaches 89.02%. In the fourth group of experiments, the best effect is that the λ function layer replaces the first, second, and fourth groups, and the CoT layer replaces the third group, and the recognition accuracy reaches 88.43%. From this, we get the optimal convolution module collocation form: the first two groups are replaced by the λ function layer, and the CoT layer replaces the last two groups. We renamed the optimal model LaCTResNet5012 as LaCTResNet50.

Next, we use the 1:1:1:1 module distribution ratio of ResNet18 and Swin Transformers’ 1:1:3:1 module distribution ratio (26) to discuss the LaCTResNet50 model further. These two ratios are proved effective in improving the model’s accuracy (23, 27), and the experimental results are shown in **Table 4**.

**Table 4:** The recognition accuracy and parameters of the models with different module ratios on the monkeypox independent test set.

Model Name	Number of modules	Accuracy	Params (M)
LaCTResNet50	[3, 4, 6, 3]	89.02%	32.65
LaCTResNet2222	[2, 2, 2, 2]	89.26%	19.95
LaCTResNet3333	[3, 3, 3, 3]	87.84%	27.86
LaCTResNet2262	[2, 2, 6, 2]	89.02%	26.14
LaCTResNet3393	[3, 3, 9, 3]	91.85%	37.14

According to the above table, the recognition accuracy of LaCTResNet2222 and LaCTResNet3393 is higher than that of LaCTResNet50, with an increase of 0.24% and 2.83%, respectively. Although the recognition accuracy of LaCTResNet2262 is the same as that of LaCTResNet50, it has certain advantages in parameters. In the end, the best model we got was LaCTResNet3393, and the recognition accuracy was 91.85% on the independent test set of the monkeypox data set. According to the overall structural characteristics of the network, we renamed LaCTResNet2222 to LaCTResNet26, LaCTResNet2262 to LaCTResNet38, and LaCTResNet3393 to LaCTResNet56.

The experimental data mentioned above show that the LaCTResNet model we designed has the advantages of both lightweight and high accuracy. In order to be able to disclose the details of the model’s framework more intuitively, we output the framework structure of the LaCTResNet series of models through the program and list the parameters in **Table 5**.

**Table 5:** Details of LaCTResNet series network parameters.

Layer Name	Output Size	LaCTResNet26	LaCTResNet38	LaCTResNet56
Conv1	112×112	7×7, 64, stride2		
LamResConv2	56×56	3×3, max pool, stride2		
		$\left(\begin{array}{c} 1\times 1,64\\ \lambda ,64\\ 1\times 1,256 \end{array}\right)\times 2$	$\left(\begin{array}{c} 1\times 1,64\\ \lambda ,64\\ 1\times 1,256 \end{array}\right)\times 2$	$\left(\begin{array}{c} 1\times 1,64\\ \lambda ,64\\ 1\times 1,256 \end{array}\right)\times 3$
LamResConv3	28×28	$\left(\begin{array}{c} 1\times 1,128\\ \lambda ,128\\ 1\times 1,512 \end{array}\right)\times 2$	$\left(\begin{array}{c} 1\times 1,128\\ \lambda ,128\\ 1\times 1,512 \end{array}\right)\times 2$	$\left(\begin{array}{c} 1\times 1,128\\ \lambda ,128\\ 1\times 1,512 \end{array}\right)\times 3$
CoTResConv4	14×14	$\left(\begin{array}{c} 1\times 1,256\\ \mathrm{CoT},256\\ 1\times 1,1024 \end{array}\right)\times 2$	$\left(\begin{array}{c} 1\times 1,256\\ \mathrm{CoT},256\\ 1\times 1,1024 \end{array}\right)\times 6$	$\left(\begin{array}{c} 1\times 1,256\\ \mathrm{CoT},256\\ 1\times 1,1024 \end{array}\right)\times 9$
CoTResConv5	7×7	$\left(\begin{array}{c} 1\times 1,512\\ \mathrm{CoT},512\\ 1\times 1,2048 \end{array}\right)\times 2$	$\left(\begin{array}{c} 1\times 1,512\\ \mathrm{CoT},512\\ 1\times 1,2048 \end{array}\right)\times 2$	$\left(\begin{array}{c} 1\times 1,512\\ \mathrm{CoT},512\\ 1\times 1,2048 \end{array}\right)\times 3$
Average Pooling	1×1	/	/	/
Fully Connected				
Softmax				

### 4.3 5-fold cross-validation

To ensure the reliability and stability of the model, we evaluated the model using cross-validation experiments. For a limited sample dataset, five-fold cross-validation is commonly used to evaluate or compare the performance of models. In 5-fold cross-validation, the dataset is divided into five mutually exclusive subsets (i.e., *D = D_1_* ∪ *D_2_* ∪… ∪ *D_5_, D_i_* ∩ *D_j_* = ∅ (*i* ≠ *j*)), where *D* - *D_i_* is used as the training set and *D_i_* (*i* = *1*, *2*,*…*, *5*) as the validation set. The cross-validation process is repeated five times, and the results of the five times are averaged to evaluate the model’s performance. The 5-fold cross-validation of our proposed model achieves an average recognition accuracy of 91.25% on the training set and 90.54% on the test set. The standard deviations of the above experimental data are minor, only 0.0091 and 0.0119. The experimental data are shown in **Table 6**.

**Table 6:** Recognition accuracy of 5-fold cross-validation.

Fold	Train Accuracy	Test Accuracy
1	0.9205	0.9185
2	0.8977	0.8863
3	0.9233	0.9168
4	0.9091	0.8992
5	0.9119	0.9064
Mean	0.9125	0.9054
Standard deviation	0.0091	0.0119

### 4.4 Contrast experiments

We selected the classic deep convolution neural networks AlexNet, VGG16, Inception-V3, ResNet50, and EfficientNet-B5 as the comparative experimental models, and the experimental results are shown in **Table 7**.

**Table 7:** The recognition accuracy and parameters of the classical network models and LaCTResNet series models in the monkeypox independent test set.

Model Name	Accuracy	Params (M)
AlexNet	66.82%	61.10
VGG16	71.19%	138.36
Inception-V3	57.85%	22.32
ResNet50	76.03%	25.56
EfficientNet-B5	75.80%	2.22
LaCTResNet26	89.26%	19.95
LaCTResNet38	89.02%	26.14
LaCTResNet50	89.02%	36.65
LaCTResNet56	91.85%	37.14

**Figure 7:**
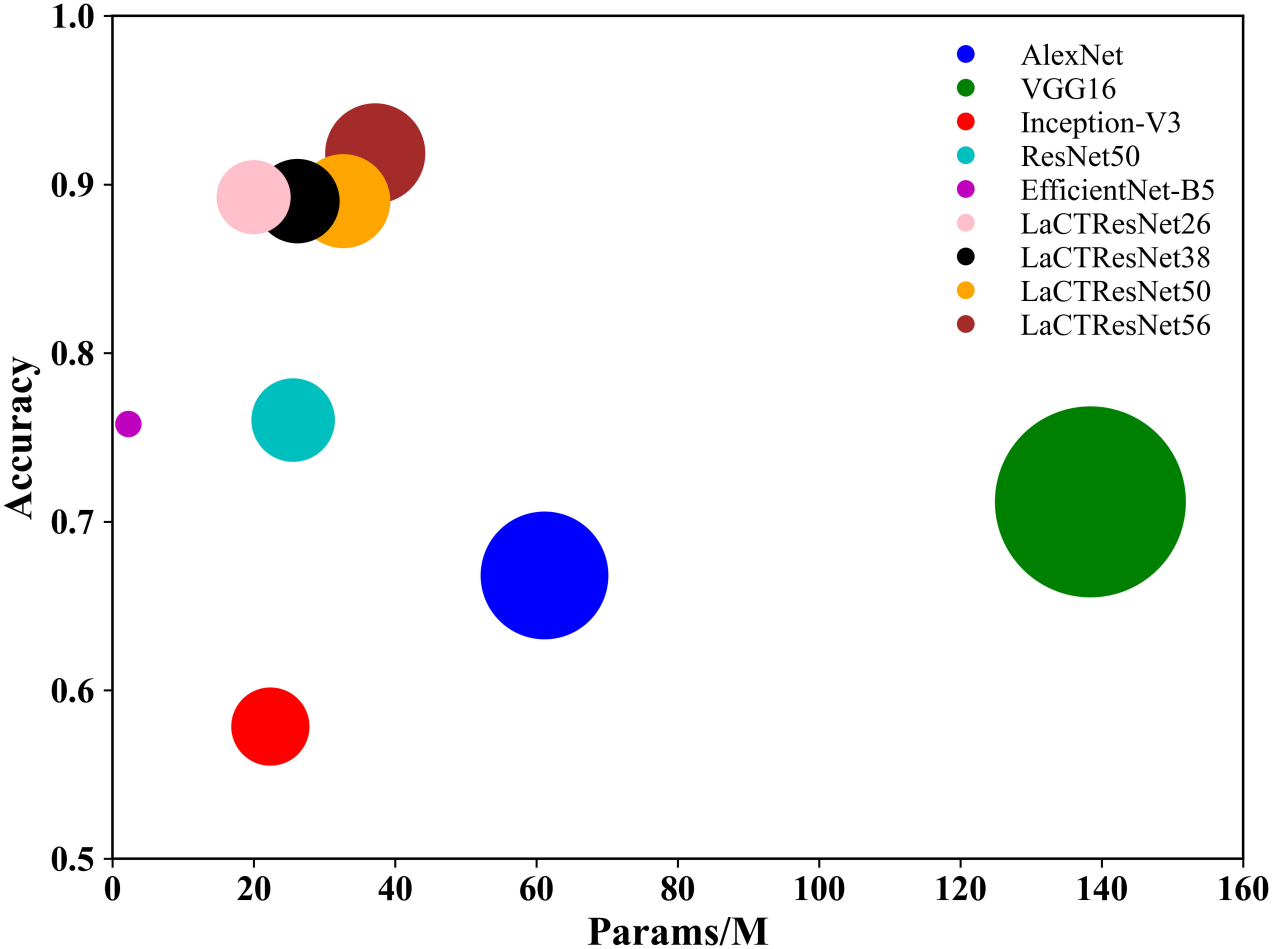
The recognition accuracy and parameter quantity of the models. The vertical coordinate indicates the average recognition accuracy of the model, and the horizontal coordinate indicates the number of parameters of the model.

The above table shows that the recognition accuracy of classical network models on the independent test set of monkeypox is all below 80%. In comparison, the recognition accuracy of the LaCTResNet series models proposed by us is about 90%, which is 13.23%, 13.03%, and 15.82% higher than that of the ResNet50 model. In order to more intuitively reflect the relationship between the accuracy and parameters of each network model, we draw the relationship diagram between accuracy and parameters, as shown in **Figure 7**, through visualization software. Considering these two parameters comprehensively, we find that LaCTResNet series models have advantages.

## 5 Results analysis and performance evaluation

### 5.1 Performance of the model on the training set

**Figure 8:**
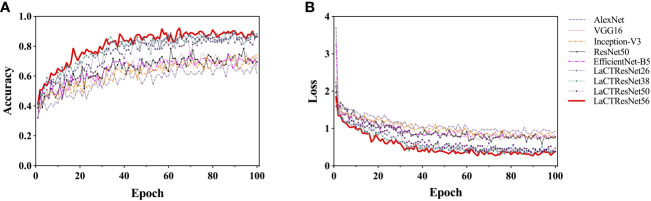
The recognition accuracy and loss curve of models on the training set. The vertical coordinates in **(A)** indicate the accuracy rate; the vertical coordinates in **(B)** indicate the loss value; the horizontal coordinates all indicate the training period.

In this section, we analyze the accuracy and convergence of the loss value of the model on the monkeypox training set in detail. In the experiment, we adopt a dynamic learning rate strategy. That is, the learning rate decays by 90% every 30 training cycles. This strategy can make the model jump out of the “trap” of optimal local value and avoid local oscillation, thus effectively improving the convergence speed of the model. The two-dimensional line chart of each model’s accuracy and loss value during training is shown in **Figure 8**. Observing all the curves in the diagram, we can find that the recognition accuracy of each model has significantly jumped after the 30th cycle, and the loss values have decreased significantly. After the 60th cycle, the accuracy and loss values gradually converged and approached a stable value. Let us compare the classic model and LaCTResNet series models as two groups. We can find that the recognition accuracy of the classic model fluctuates between 0.5 and 0.8 after the 60th cycle, while that of LaCTResNet series models fluctuates between 0.7 and 0.9. LaCTResNet series models have apparent advantages in convergence speed and recognition accuracy. The overall trend of the accuracy and loss value curve is consistent with the target expectation and fluctuates in the normal range, showing that the model can deal with local optimal traps, performs well in over-fitting, and can fully grasp the potential “universal law” in the sample.

### 5.2 Performance of the model on the independent test set

**Figure 9:**
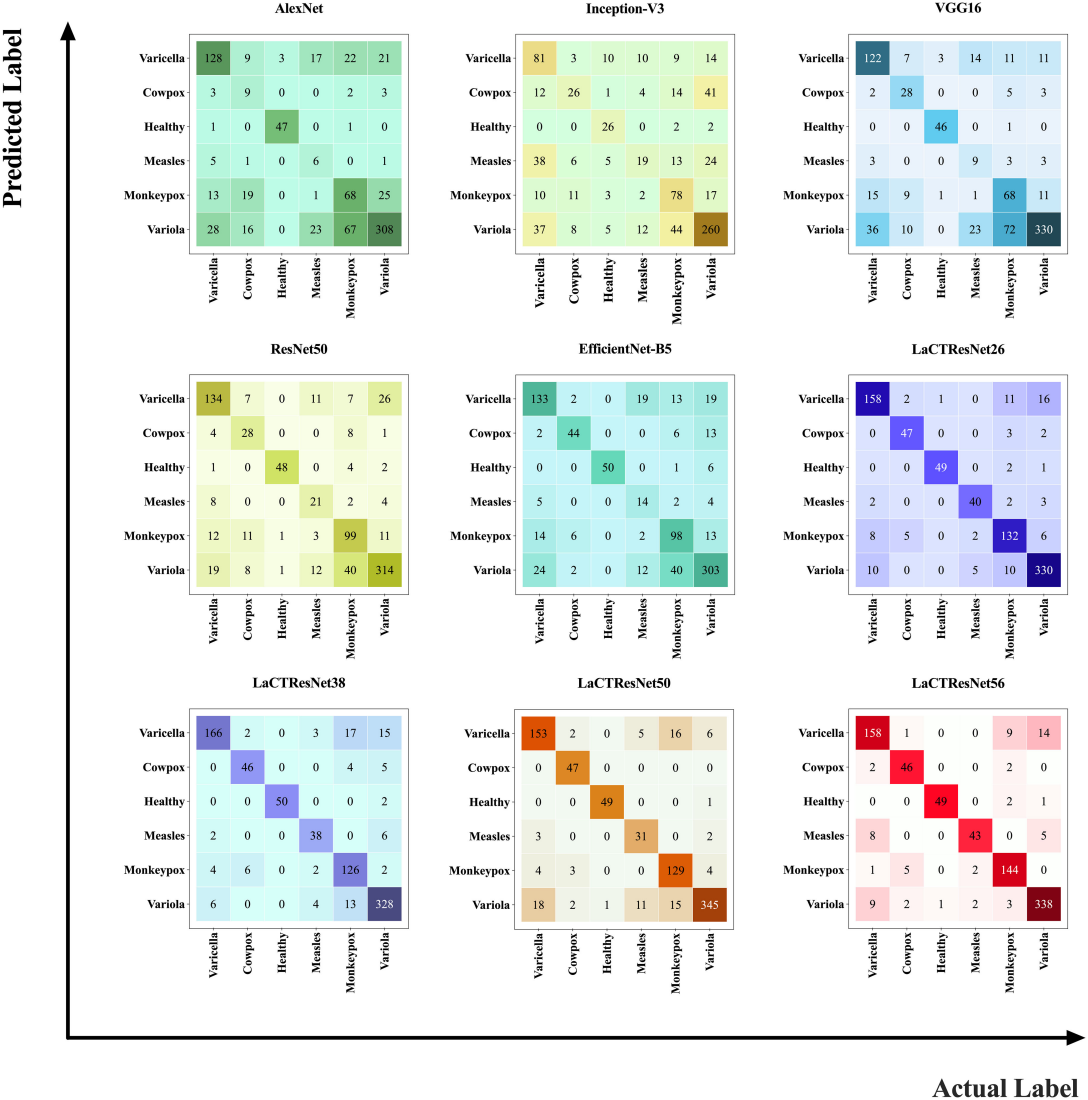
Confusion matrix heat map. The number represents the total number of sample images predicted as that class by the model; the larger the value, the darker the color.

In order to verify the generalization ability and robustness of the model, we conducted prediction experiments on the LaCTResNet series model on an independent test set of monkeypox images, and the confusion matrix heat map was drawn by a visualization tool, as shown in **Figure 9**, which can visually demonstrate the recognition ability of the model.

In the heat map, the vertical coordinates represent the predicted values of the samples, and the horizontal coordinates represent the actual values of the samples. The main diagonal numbers represent the number of images when the predicted values agree with the actual values, i.e., the number of images correctly recognized by the model, and the remaining position numbers represent the number of images when the predicted values differ from the actual values, i.e., the number of images incorrectly recognized by the model. Observing the confusion matrix heat map of all models, we can find that the classical model has a significant difference in the color depth of the main diagonal blocks of the heat map, which indicates that the model incorrectly recognizes more images; the main diagonal blocks of the heat map of the LaCTResNet series of models all consistently show a darker color, which indicates that the series of models proposed in this paper show better recognition of all six types of images on the monkeypox data set. It has certain technical reference value and application significance.

The model’s performance can be evaluated more quantitatively by further analyzing and processing the values in the confusion matrix. In this regard, we must also define the following metrics: *TP*, *FP*, *TN*, and *FN*, representing true positive, false positive, true negative, and false negative, respectively. Among them, *Precision* is the proportion of positive cases correctly predicted by the model to the actual positive cases, reflecting the checking accuracy of the model, and the mathematical expression is shown in equation (11):


(11) $$Precision=\frac{TP}{TP+FP};$$

*Recall*, also known as True Positive Rate (*TPR*), refers to the proportion of positive cases correctly predicted by the model to all positive cases predicted by the model, reflecting the model’s check-all rate and the mathematical expression is shown in equation (12):


(12) $$Recall=TPR=\frac{TP}{TP+FN};$$

The False Positive Rate (*FPR*) refers to the proportion of positive cases that the model incorrectly predicts to all positive cases, also called the false identification rate and false alarm rate, and the mathematical expression is shown in equation (13):


(13) $$FPR=\frac{FP}{TN+FP};$$

The F1 Score (*F1_score*) is the summed average of precision and recall, which is used to measure the comprehensive performance of the model and the mathematical expression is shown in equation (14):


(14) $$F1\_score=\frac{2\times Precision\times Recall}{Precision+Recall}$$

Since this experiment is a multiclassification problem, the macro average of the above evaluation metrics is used to measure the “global” performance of the model, which are the macro accuracy rate (*macro-P*), macro completeness rate (*macro-R*) and macro F1 score (*macro-F1_score*), and the mathematical expressions are shown in equations (15), (16) and (17), respectively:


(15) $$macro-P=\frac{1}{n}\sum_{i=1}^{n}P_{i},$$

(16) $$macro-R=\frac{1}{n}\sum_{i=1}^{n}R_{i},$$

(17) $$macro-F1=\frac{2\times macro-P\times macro-R}{macro-P+macro-R},$$

Where, *P_i_* refers to the precision of the *i*th class; *R_i_* refers to the recall of the *i*th class. Detailed data on the recognition performance of the LaCTResNet family of models are shown in **Table 8**.

**Table 8:** Accuracy, Recall, and F1 Score of each category.

	Model Name	Varicella	Cowpox	Healthy	Measles	Monkeypox	Variola	Average
Precision	LaCTResNet26	0.8404	0.9038	0.9423	0.8511	0.8627	0.9296	0.8883
	LaCTResNet38	0.8177	0.8364	0.9615	0.8261	0.9	0.9345	0.8794
	LaCTResNet50	0.8407	1	0.98	0.8611	0.9214	0.8801	0.9139
	LaCTResNet56	0.8681	0.92	0.9423	0.7679	0.9474	0.9521	0.8996
Recall/TPR	LaCTResNet26	0.8876	0.8704	0.98	0.8511	0.825	0.9218	0.8893
	LaCTResNet38	0.9326	0.8519	1	0.8085	0.7875	0.9162	0.8828
	LaCTResNet50	0.8596	0.8704	0.98	0.6596	0.8062	0.9637	0.8566
	LaCTResNet56	0.8876	0.8519	0.98	0.9149	0.9	0.9441	0.9131
F1_score	LaCTResNet26	0.8634	0.8868	0.9608	0.8511	0.8434	0.9257	0.8885
	LaCTResNet38	0.8714	0.8441	0.9804	0.8172	0.84	0.9253	0.8797
	LaCTResNet50	0.85	0.9307	0.98	0.747	0.86	0.92	0.8813
	LaCTResNet56	0.8777	0.8846	0.9608	0.835	0.9231	0.9481	0.9049
FPR	LaCTResNet26	0.0448	0.0063	0.0038	0.0088	0.0306	0.0511	/
	LaCTResNet38	0.0553	0.0113	0.0025	0.01	0.0204	0.047	
	LaCTResNet50	0.0433	0	0.0013	0.0063	0.016	0.0961	
	LaCTResNet56	0.0359	0.005	0.0038	0.0163	0.0116	0.0348	

Observing the information in the above table, we can find that the LaCTResNet56 model has the most significant *macro-F1_score* value as well as the highest *F1_score* value for monkeypox case recognition, the model has the best overall recognition performance, and the overall recognition of monkeypox cases is better than other models.

**Figure 10:**
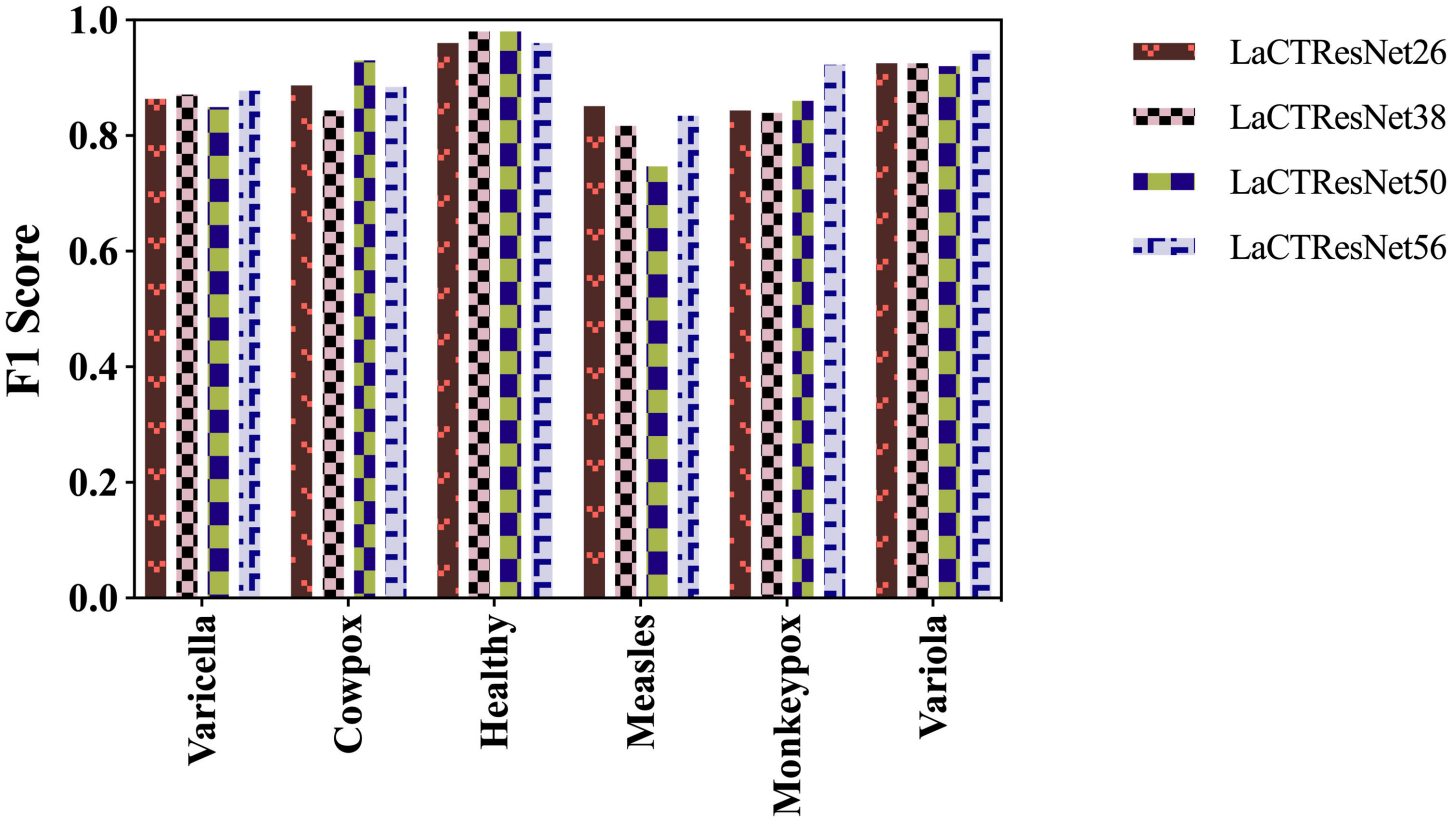
F1 Score of the LaCTResNet family of models.

As shown in **Figure 10**, the combined recognition ability of the LaCTResNet family of models for each category is demonstrated. The models are less effective in recognizing measles cases. It is because the clinical symptoms of measles, chickenpox, and smallpox all show a large rash on the skin with redness and swelling. The characteristics are highly similar, which can easily lead to model misidentification.

**Figure 11:**
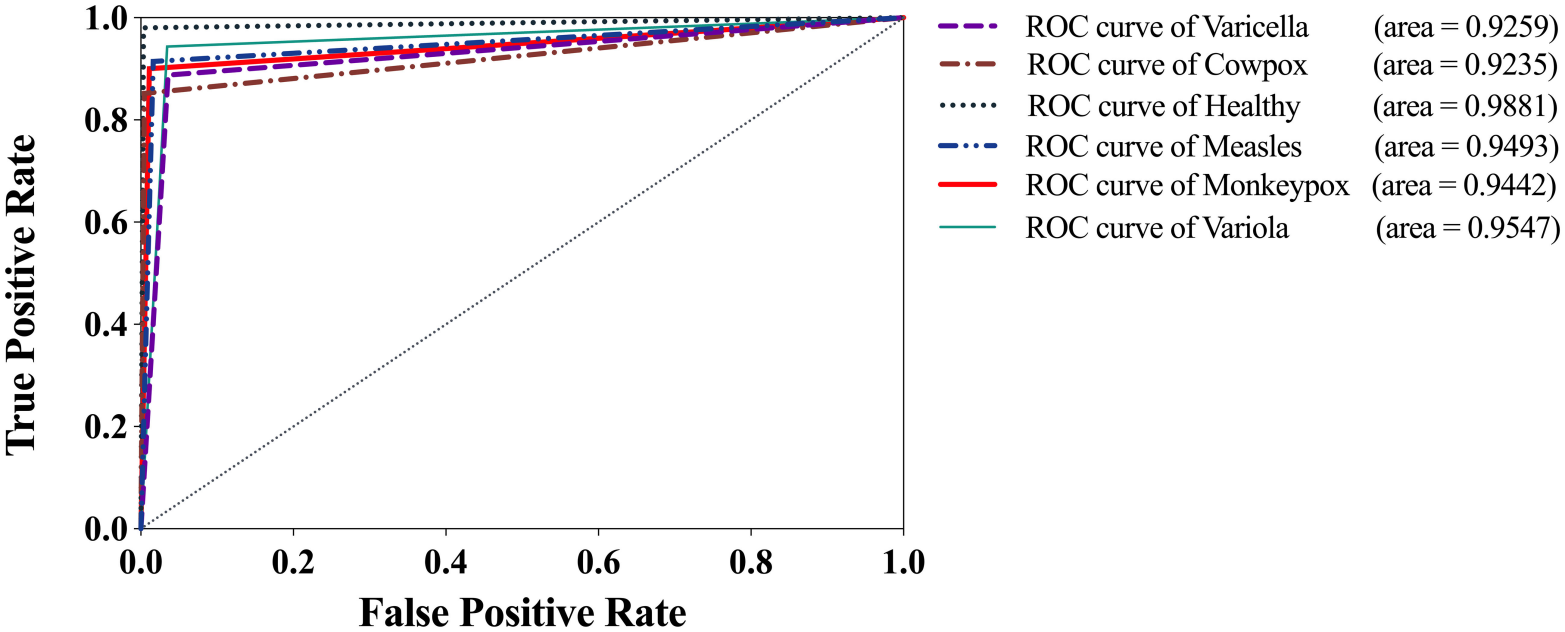
ROC curve of LaCTResNet56 model.

**Figure 11** shows the Receiver Operating Characteristic (ROC) curve of the optimal model, LaCTResNet56, for each category of cases on the monkeypox dataset. The vertical coordinate represents the *TPR*, the horizontal coordinate represents the *FPR*, and the Area Under Curve (AUC) represents the recognition effect of the model for that category, and the more significant the area, the better the effect. If the AUC is less than 0.5, it means that the model does not have realistic reference significance for identifying this category, while the closer the AUC is to 1, the better the model is for identifying this category. It can be seen from the figure that the AUC of measles cases is 0.9235. It is worth mentioning that the AUC of monkeypox cases reaches 0.9442, and the average AUC of all categories reaches 0.9476, proving that the model can identify monkeypox cases with high accuracy.

## 6 Conclusions

In this study, we introduced the λ function layer and CoT function layer to replace the 3×3 convolutional layer in the original model based on the original ResNet model and defined a new residual convolution module. We tried to replace all the original convolution modules with new modules containing CoT function layers. The accuracy of this model on the dataset reached 84.06%. By replacing the first module with new modules containing λ function layers and the rest with new modules containing CoT function layers, the accuracy of this model on the dataset reached 87.37%. Based on the good results of the above experiments, we continued to optimize the model structure by discussing the position of the two new modules and the ratio of the number of new modules, respectively and finally found that the model constructed by replacing the first two modules with modules containing the λ function layer, and replacing the last two modules with modules containing the CoT function layer, and using either a 1:1:1:1 ratio of the modules or a 1:1:3:1 ratio, was able to exhibit excellent performance. Among them, our constructed optimal model LaCTResNet56 achieves an average recognition accuracy of 91.85% on the test set, which is 15.82%, 7.79%, and 29.89% better than the baseline models ResNet50, CoTResNet50, and LambdaResNet50, and better than the similar models AlexNet, VGG16, Inception-V3, and EfficientNet-B5 by 25.03%, 20.66%, 34.00%, and 16.05%, respectively. We evaluated the recognition accuracy of the optimal model using the ROC curve and concluded that the model has a strong recognition ability for monkeypox cases, with an AUC of 0.9442. The above experimental data aim to show that our model has excellent comprehensive performance, can effectively extract the feature information of monkeypox cases and identify similar cases efficiently and reliably, and has certain practical significance in the auxiliary diagnosis of monkeypox cases.

Our future work is mainly based on the following considerations: (1) To collect as many images of monkeypox clinical cases as possible and continuously optimize the model. (2) Since there are already mutated strains of the monkeypox virus, we will collect clinical images to classify cases of different monkeypox strains individually and conduct experiments to support the precision of monkeypox epidemic tracking. (3) The model will be deployed to intelligent devices such as cell phones.

## Data availability statement

Publicly available datasets were analyzed in this study. This data can be found here: https://www.kaggle.com/datasets/maxmelichov/monkeypox-2022-remastered.

## Ethics statement

The studies involving humans were approved by ethics committee of the Experimental Animal Center of Gansu Agricultural University(GSAU-Eth-ASF2022-008). The studies were conducted in accordance with the local legislation and institutional requirements. Written informed consent for participation was not required from the participants or the participants’ legal guardians/next of kin because Our experiment used a public dataset (see: https://www.kaggle.com/datasets/maxmelichov/monkeypox-2022-remastered). We did not have direct contact with patients to collect clinical data. Anyone who may be identified in the dataset has been blindfolded.

## Author contributions

Conceptualization, JC and JH; Data curation, JC and JH; Formal analysis, JC; Funding acquisition, JH; Investigation, JC; Methodology, JC; Project administration, JC; Resources, JC and JH; Software, JC; Supervision, JC; Validation, JC and JH; Visualization, JC; Writing—original draft, JC; Writing—review and editing, JC and JH. All authors contributed to the article and approved the submitted version.
